# UPLC-MS/MS Phytochemical Analysis of Two Croatian *Cistus* Species and Their Biological Activity

**DOI:** 10.3390/life10070112

**Published:** 2020-07-14

**Authors:** Ivana Carev, Ana Maravić, Nada Ilić, Vedrana Čikeš Čulić, Olivera Politeo, Zoran Zorić, Mila Radan

**Affiliations:** 1 Faculty of Chemistry and Technology, University of Split, 21000 Split, Croatia; ivana.carev@ktf-split.hr (I.C.); olivera@ktf-split.hr (O.P.); 2 Faculty of Science, University of Split, 21000 Split, Croatia; amaravic@pmfst.hr (A.M.); nada.ilic.pekic@gmail.com (N.I.); 3 School of Medicine, University of Split, 21000 Split, Croatia; vcikesc@mefst.hr; 4 Faculty of Food Technology and Biotechnology, University of Zagreb, 10000 Zagreb, Croatia; zzoric@pbf.hr

**Keywords:** antimicrobial, antioxidant, antiproliferative, *Cistus*, flavonoid, UPLC-MS/MS

## Abstract

Aqueous extracts of two *Cistus* species wild growing in Croatia—*Cistus creticus* (CC) and *Cistus salviifolius* (CS)—have been assessed with UPLC-MS/MS, showing 43 different phytochemicals, with flavonol glycosides: myricetin-3-hexoside and myricetin-rhamnoside, predominate ones in CC and myricetin-3-hexoside in CS. Antioxidant potential tested with the FRAP method showed no difference between CS and CC aqueous extracts, while higher phenolic content of CC comparing to CS, determined with a Folin–Cicolateu reagent correlated to its higher antioxidant capacity observed by the DPPH method. Both extracts were assessed for antimicrobial activity, using disc-diffusion and broth microdilution assays, targeting the opportunistic pathogens, associated with food poisoning, urinary, respiratory tract, blood stream and wound infections in humans. Antimicrobial assays revealed that fungi were in general more sensitive to both *Cistus* aqueous extracts, comparing to the bacteria where two extracts showed very similar activity. The most potent activity was observed against *A. baumannii* for both extracts. The extracts were tested on human lung cancer (A549) cell line using the MTT assay, showing very similar antiproliferative activity. After 72 h treatment with CC and CS aqueous extracts in concentration of 0.5 g/L, the viability of the cells were 37% and 50% respectively, compared to non-treated cells.

## 1. Introduction

The *Cistus* genus belongs to a small family of Cistaceae of eight genera, widely distributed in the Mediterranean. The species are known as rock roses and have noteworthy morphological diversification. This genus has several medicinal perennial shrubs widely distributed in the Mediterranean area [1].

Since ancient times, Cistaceae have been used in ethnomedicine due to their pharmacological potential against a broad range of disorders, including various skin diseases, diarrhea, ulcers, dysentery, catarrh, menstruation difficulties, and gout due to a number of natural pharmacological compounds they consist of [2,3,4]. According to ethno-pharmacology, the *Cistus* species has been used due to its antimicrobial, antiproliferative, anticancer, antiviral, antioxidant, anti-inflammatory, antiulcerogenic, antidiarrheal, and antispasmodic activity [5,6,7,8,9,10,11,12,13,14,15,16,17,18,19,20,21,22,23,24,25,26]. It is also known that some *Cistus* species have been used in human and animal diets, such as goats, lamb and beef [27,28,29,30,31,32,33,34].

Phytochemical profile of different *Cistus* species aqueous extracts reveals their polyphenolic profile, having flavonoids, phenolic acids, and ellagitanins as their main constituents [2,3,6,8,10,15,23,26,31,32,35,36,37,38,39,40,41,42,43,44].

This study examined two wild growing Croatian *Cistus* species, *Cistus salviifolius* (CS) and *Cistus creticus* (CC), known to be used in herbal tea preparation in human diet [34]. To the best of our knowledge this is the first UPLC-MS/MS phytochemical phenolic profile analysis of *C. creticus* and *C. salvifolius* aqueous extract. Due to the traditional use of *Cistus* species in human and animal diet, as well as for their pharmacological potential in ethno-medicine, we have examined the biological activity of CC and CS aqueous extracts. The pharmacological potential of two *Cistus* aqueous extracts was tested against the emerging opportunistic pathogens associated with skin, nail, gastrointestinal, bloodstream and respiratory infections, as well as their in vitro antiproliferative activity, antioxidant potential and total phenolic content.

## 2. Materials and Methods

### 2.1. Plant Material

Plant material, the upper part, stems, leaves, and flowers, of two species of the genus *Cistus* (rock rose)—*Cistus salviifolius* L. (sage-leaved rock rose), *Cistus creticus* L. (pink rock rose)—were collected on the island of Čiovo (Split, Croatia) in June 2015 and identified by a botanist, Jure Kamenjarin, Faculty of Science, University of Split. Voucher specimens of plant materials were deposited in the Department of Biochemistry, Faculty of Chemistry and Technology, Split, Croatia with numbers CS_2015_01 and CC_2015_01. Plant material was air-dried at room temperature for 3 days and 20 g of the dried material was chopped and extracted with 0.15 L of hot distilled water. Water solution was filtered after 30 min and water extracts were subjected to water evaporation by a low vacuum using the rotary evaporator. Dried aqueous extract was dissolved in distilled water and kept in a fridge at a temperature of −20 °C for further assays.

### 2.2. UPLC-MS/MS Phytochemical Analysis of Plant Extracts

Standard preparation for UPLC-MS/MS analysis was made as follows: stock standard solutions of each phenolic standard were prepared by dissolving in methanol at concentration of 0.1 g/L. Multi compound standard solutions were prepared in methanol, and dilutions from this solution were done in the range 0.00001–0.1 g/L for external calibration and validation experiments of the UPLC-MS/MS system. The three injections per level were done and peak area of each standard were used to make the respective standard curves. UPLC analyses of aqueous extracts were performed on Agilent 1290 RRLC instrument (Agilent Technologies, Santa Clara, CA, USA) coupled with binary gradient pump, autosampler, and column compartment. The gradient conditions were set up according to the methods reported [45]. The column used for separation was Zorbax Eclipse Plus C18 column (100 × 2.1 mm, 1.8 µm) (Agilent, Santa Clara, CA, USA).

Mass spectrometry experiments and optimization of the method were performed using a triple quadrupole mass spectrometer (QQQ 6430, Agilent, Santa Clara, CA, USA). The mass spectrometer was used in the dynamic multiple reaction monitoring mode (dMRM) in the ESI-positive and negative mode and operated with the following source parameters: capillary voltage, +4000/−3500 V, nitrogen drying gas temperature maintained at 300 °C with a flow rate of 11 L/h and the pressure of the nebulizer was set at 40 psi. Mass hunter software was used for data acquisition and analysis. Identification of the phenolic compounds was performed by comparing retention times and mass spectra with those of the authentic standards. In case of unavailability of standards, the prediction and structural identification of phenolic compounds was carried out by comparing the mass fragments with the previously reported mass fragmentation patterns [42,46]. Analytical method quality parameters, such as calibration curve, instrumental detection (LOD) and quantification (LOQ) limits were determined as previously reported by Zorić [45]. Quantitative data for all phenolic compounds were obtained by calibration curves of known standards. If a commercial standard was not available, quantification was performed using the calibration curve of standards from the same phenolic group. Results are shown as the mean values with the standard deviation.

### 2.3. DPPH Test

The antiradical activity of *Cistus* aqueous extracts was determined using the DPPH (2,2′-diphenyl-1-picrylhydrazy) radical scavenging method [47]. The antiradical activities of the samples were calculated according to the formula:

% inhibition = [(A_0_ − A_sample_)/A_0_] × 100, (1)

where A_0_ was absorbance of the DPPH ethanolic solution measured at the beginning and A_sample_ was absorbance of the sample measured after 60 min. The results were expressed as IC_50_ or the concentration of the extract that gives 50% of the inhibition of DPPH radical reaction.

### 2.4. FRAP Assay

The reducing antioxidant power of two *Cistus* aqueous extracts was determined using the FRAP method [48]. FRAP assay measures the ability of plant extracts to reduce the ferric 2,4,6-tripyridyl-s-triazine complex [Fe(III)–(TPTZ)2]2þ to the intensely blue colored ferrous complex [Fe(II)–(TPTZ)2]2þ in acidic medium. Reducing power of samples was calculated comparing the reaction signal given with solution of Fe^2+^ ions in known concentrations and expressed as mM equivalents of Fe^2+^ ions.

### 2.5. Total Phenolic Content

Total phenolic content was measured spectrophotometrically according to the Folin–Ciocalteu colorimetric method [49]. Gallic acid (Sigma-Aldrich, Steinheim, Germany) was used as a standard and samples were taken in triplicate, while the results were expressed as gallic acid equivalents.

### 2.6. MTT Assay

Lung cancer cell line A549 was grown at 37 °C in a humidified incubator and 5% CO_2_ in Dulbecco’s Modified Eagle Medium (DMEM, EuroClone, Milano, Italy) containing 10% fetal bovine serum and 1% antibiotics (Penicillin Streptomycin, EuroClone, Milan, Italy). Cell viability was determined using the tetrazolium salt (3-[4,5-dimethylthiazol-2-yl]-2,5-diphenyl tetrazolium bromide; MTT Sigma-Aldrich) in reduction MTT assay (Hussain, Nouri, and Oliver, 1993). An equal number of cells were seeded into the wells of a 96-well plate and allowed to attach overnight. Cells were afterward treated with both extracts (100 μL), in triplicate concentrations of 0.5 g/L; 1 g/L; and 2 g/L in growing media for 4 h, 24 h, 48 h, and 72 h. Following treatment, cells were incubated with 0.5 g/L MTT in growing media for 1 h. After media was removed, DMSO (Sigma-Aldrich) was added to the cells. Absorbance was measured at 570 nm (signal) and 690 nm (background).

### 2.7. Determination of the Antimicrobial Activity 

The antimicrobial screening of two *Cistus* aqueous extracts was carried out using disc-diffusion and broth microdilution assays according to the guidelines of the Clinical Laboratory Standards Institute [50,51].

For preliminary screening, a disc diffusion assay was employed. Briefly, an aliquot of 100 µL of suspension of the mid-exponentially grown bacteria containing 106 CFU/mL of cells, and 0.5–2.5 × 10^3^ CFU/mL of *C. albicans* spores were plated on MHA (Biolife) and SDA (Biolife). The 6 mm sterile filter discs (BectonDickinson, Franklin Lakes, NJ, USA) were individually loaded with 50 µL of the stock solution of *Cistus* aqueous extracts (0.1 g/mL) equivalent to a final concentration of 0.005 g/disc and then placed on the agar surface previously inoculated with the microbial strains. Tetracycline (30 µg) and amphotericin B (10 µg) discs were used as positive controls. The plates were incubated for 24 h at 37 °C. The antimicrobial activity was determined by measuring the diameters of the inhibition zones in millimeters. Assays were done in triplicate and the obtained values expressed as mean ± standard error (SE).

For the microdilution assays, experiments were performed in 96-well microtiter plates with serial dilutions of extracts ranging from 32 to 0.03125 mg/mL. The mid-exponentially grown bacterial cultures were adjusted spectrophotometrically to achieve optical densities corresponding to 1 × 10^6^ CFU/mL, and 0.5–2.5 × 10^3^ CFU/mL in case of *C. albicans*. After 24 h of incubation at 37 °C, the minimal inhibitory concentration (MIC) was recorded as the lowest concentration of the extract showing no visually detectable microbial growth in the wells.

For *C. albicans*, the corresponding aliquots from the wells were plated on SDA and incubated for 24 h. After visual inspection and colony counting, MIC90 endpoints were recorded as the lowest concentration, inhibiting 90% of fungal growth when compared to the control-strain grown without the extract.

### 2.8. Microbial Strains

The antimicrobial activity of aqueous *Cistus* extracts were assayed against eleven microbial strains, including the most emerging Gram-positive and Gram-negative pathogens: the type strains from the American Type Culture Collection (ATCC, Rockville, MD, USA) and the multidrug-resistant (MDR) hospital strains of *Staphylococcus aureus, Klebsiella pneumoniae, Acinetobacter baumannii*, and *Pseudomonas aeruginosa*. The MDR strains demonstrated resistance to antimicrobial agents from at least three different classes, particularly to the *β*-lactam family of antibiotics as a result of *β*-lactamase(s) production (Table 1). The antibiotic susceptibility testing was assessed by Etest (AB Biodisk, Solna, Sweden) and the VITEK 2 system (bioMérieux, Marcy-l’Étoile, France). The molecular detection of genes mediating the *β*-lactam resistance phenotype was exerted as previously published [52]. Antibacterial screening also included the environmental strains of foodborne pathogens *Bacillus cereus* and *Clostridium perfringens*. The antifungal activity was assessed on the environmental strain of opportunistic pathogenic yeast *Candida albicans* (Table 2). All the microbial strains were stored at −80 °C in glycerol stocks and sub-cultured on tryptic soy agar (Biolife, Milan, Italy) or Sabouraud dextrose agar (Biolife, Milan, Italy) in case of fungi prior to testing.

## 3. Results and Discussion

### 3.1. UPLC-MS/MS Phytochemical Analysis of Plant Extracts

The preliminary phytochemical analysis of two *Cistus* aqueous extracts were performed using UPLC-MS/MS. Results of 43 chemical compounds along with their retention time, precursor ion, product ion, collision energy, and ionization mode optimized for each phenolic compound are presented in Table 1. A total of 43 polyphenolic compounds were identified, using standards where available. If a commercial standard was not available, quantification was performed using the calibration curve of standards from the same phenolic group.

Based on UPLC-MS/MS profiling of two *Cistus* extracts, this study reveals that aqueous extracts of CC and CS represent a rich source of polyphenols with flavonol derivatives being the most abundant compounds in both samples. Two flavonol glycosides, myricetin-3-hexoside and myricetin-rhamnoside, predominate in CC aqueous extracts. Myricetin-3-hexoside predominates in CS too, while myricetin-rhamnoside is present in significantly smaller amounts. Besides these two dominant phenolic compounds, two of the studied species show general similarity in the content for most of the analyzed phenolics, except that CC has a significantly higher amount of myrcetin-rutinoside, myrcetin, quercetin-rhamnoside, and procyanidin B2, while CS has a higher amount of quercetin-3-glucoside, quercetin-pentoside, kaempferol-3-glucoside, kaempferol-3-rutinoside, kaempferol-rhamnosyl-hexoside, and gallic acid, when compared to CS.

The phenolic profile of both extracts comprises phenolic acids, flavanols, flavonol glycosides and flavonones, which is in accordance with results obtained by previous *Cistus* studies. This is the first study of the phenolic composition in C. certicus and C. salvifolius aqueous extracts and the presence of phenolic compounds is in accordance with previous studies related to phenolic content in other *Cistus* species [11,42,53,54,55,56].

### 3.2. Antioxidant Activity of Cistus Extracts

The total phenolic content and antioxidant activity of CC and CS were assessed using in vitro assays and the results for both extracts are shown in Table 2.

The higher phenolic content of CC (209.27 mg of GAE/g) and CS (161.09 mg of GAE/g) aqueous extracts were at least doubled than in the previous study on the same species from Syria [57]. Higher phenolic content of CC (209.27 mg of GAE/g) in respect to CS (161.09 mg of GAE/g) aqueous extract determined through the Folin-Ciocalteu reagent correlates to its higher antioxidant capacity observed with the DPPH method (IC_50_ values were 24.0 μg/mL and 29.5 μg/mL, respectively). Correlation between phenolic content and antioxidant activity measured by DPPH method is in accordance with the fact that both assays are based on the electron-transfer mechanism [58]. The results obtained for DPPH measurements correlate with the literature data reported for three Tunisian′s *Cistus* [59]. Similar findings are also reported for CC and CS extracts from Syria with IC_50_ values of 11 μg/mL and 19 μg/mL, respectively [57]. The FRAP assay showed no positive correlation between antioxidant activity and total phenolic content in this study. The results obtained for the FRAP measurements for CC and CS were 0.78 Eq mM Fe^2+^ for both extracts.

The values obtained for antioxidant measurements of two *Cistus* extracts using DPPH and FRAP are consistent with others reported for the *Cistus* genus [42]. Too few studies have reported and stressed CC and CS phenolic and antioxidant potential. However, *Cistus* species have already been identified as a promising source of natural antioxidant compounds from plants, and as a potential means of finding new sources of natural antioxidants, functional foods, and nutraceuticals [60,61].

In this study, the water was used as an extraction solvent to isolate hydrophilic antioxidants from the plants. Certainly, for use as food and nutraceuticals, aqueous plant extracts are nutritionally more relevant and would have obvious advantages in relation to certification and safety [60]. Comparing to other studies of *Cistus* species, this study of CC and CS aqueous extracts from Croatia showed a significant amount of determined phenolics. Since they are involved in many biological processes, the phenolic and flavonoids compounds have been the subject of numerous studies, especially in strategies of reducing or preventing the generation of oxidative stress. Besides their direct antioxidant effects, a review of recent studies on the protective role of polyphenols on human health reveals the importance of their indirect effect, too. Along with antioxidant activity, phenolic compounds have been recognized by their antimicrobial and antiproliferative properties [62,63].

### 3.3. Antiproliferative Activity of Cistus Extracts

**Figure 1:**
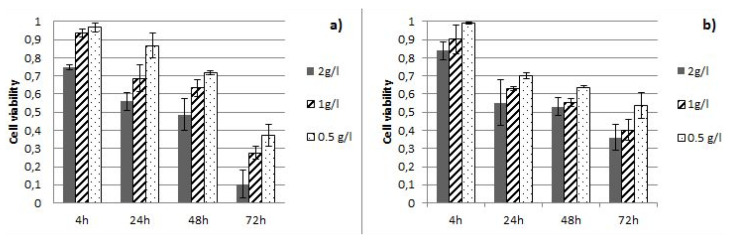
Dose-time response effect of *C. creticus* (**a**) and *C. salviifolius* (**b**) aqueous extracts on human lung cancer cell lines A549, using the MTT assay.

Antiproliferative activity of CC and CS aqueous extracts were tested on human lung cancer (A549) cell line after 4 h, 24 h, 48 h, and 72 h treatment, using the MTT assay. Both CC and CS aqueous extracts show a dose- and time-dependent antiproliferative activity against tested cell line (Figure 1).

Both *Cistus* extracts showed very similar antiproliferative activity on the lung cancer cell line A549. After 72 h of treatment with CC and CS aqueous extracts in concentration of 0.5 g/L, the cell viability was 37% and 50% respectively, compared to non-treated cells (100% of viability, cell survival = 1, Figure 1). The highest antiproliferative activity of 90% inhibition was demonstrated by CC extract after 72 h treatment for concentration of 2 g/L.

Natural herbal extracts have already been identified as a potential dietary source of polyphenols in the prevention/treatment of human chronic diseases such as cancer [64]. The studies of anticancer activity of several tea extracts have reported CC_50_ values within the range 0.1–0.5 mg/mL [65]. It has been reported that *Cistaceae* aqueous extracts containing ellagitannins showed a significant capacity to inhibit the proliferation on several cancer cell lines [66]. The results obtained in this study are in correlation with previous studies related to cytotoxicity of *Cistus* extracts, with reported CC_50_ values around 0.55 mg/mL [66,67]. The chemo-preventive and anti-tumor effects of a polyphenols-rich diet used to be associated with their ability to inhibit reactive oxygen species (ROS). However, the recent studies bring the evidence that their direct antiproliferation activity is related to modulation of uncontrolled proliferation pathways or proto-oncogene expression via multiple mechanisms [68]. Based on the fact that both extracts are rich in flavonol derivatives and that they exhibit similar antiproliferative activity, it can be assumed that these compounds can be attributed to this effect. This assumption can be supported by other studies that show the antiproliferative activity of flavonoids [69,70,71].

The present preliminary study, for the first time, reported antiproliferative activity of the *Cistus* aqueous extracts on the A549 lung cancer cell line. The concentration of 0.5 mg/mL of two *Cistus* extracts showing the antiproliferative effect against A549 cancer cells might be very significant to support further studies.

### 3.4. Antimicrobial Potential of Cistus Extracts

This study has assessed the antimicrobial potential of the CC and CS aqueous extracts, using disc-diffusion and broth microdilution assays, targeting the opportunistic pathogens that are commonly associated with food poisoning as well as urinary, respiratory tract, blood stream, and wound infections in humans. The study also included the antibiotic-susceptible ATCC strains as well as multiple resistant clinical strains of *S. aureus* (MRSA-1), *K. pneumoniae*, *A. baumannii*, and *P. aeruginosa* to observe the possible difference in the activity of extracts towards the strains of the same bacterial species but different antibiotic susceptibility/resistance patterns.

Overall, the antimicrobial assays revealed that fungi were in general more sensitive to both *Cistus* aqueous extracts in comparison to the bacteria as presented in Table 3 and Table 4.

As shown in Table 3, CS extract exhibited very good antifungal activity against opportunistic yeast C. albicans (MIC 125 µg/mL and inhibition zone of 23.2 ± 1.2 mm). On the other hand, MIC values for both extracts against bacterial strains were recorded in the range of 250 to 2000 µg/mL, respectively. The measured inhibition zones for 5 mg of each extract/disc ranged from 10.0 ± 0.5 to 29.0 ± 0.8 mm. It is important to note that, in general, the two extracts showed very similar activity towards the same bacterial species. However, slightly better activity of CC was evident against *S. aureus* ATCC 29213 and *P. aeruginosa* ATCC 27853 (MICs 500 µg/mL). Among the tested bacterial strains, the most potent activity was observed against *A. baumannii* FSST-20 clinical isolate (MIC 250 µg/mL) for both extracts. Notably, this particular multiple resistant strain as well as the *P. aeruginosa* FSST-21 and the MRSA-1 strain were found to be more sensitive and had a 2-fold reduction in MIC of at least one extract when compared to the antibiotic-susceptible ATCC strains. This finding indicates the potential of *Cistus* extracts to act against both Gram-negative and Gram-positive multiple resistant opportunistic pathogens that cause infections, which are therefore hard to treat by conventional antibiotics. The most promising activity was recorded by the disc-diffusion assay against the MRSA-1 strain, which was inhibited by 29.0 ± 0.8 and 25.0 ± 1.0 mm at the concentration of 5 mg/disc for CC and CS aqueous extracts, respectively.

The antimicrobial effect of various extracts, including the aqueous ones, derived from plants of the Cistaceae family have long been in the focus of scientific interest [72]. Most recently, *C. incanus* herbal tea has demonstrated antibacterial and anti-adherent effects against *Streptococcus mutans*, a causative agent of caries disease [34]. Moreover, *C. ladanifer* and *C. populifolius* leaf aqueous extracts were found to be active against *S. aureus* and *E. coli* (MIC50 from 0.123 to 0.9 mg/mL) [66]. However, there are only few reports on the investigation of the antimicrobial potency of CC and CS aqueous extracts. Previously, aqueous extracts obtained via spray-drying from Spanish CS were found to exhibit significant bacteriostatic and bactericidal effects against *S. aureus* CECT 59 strain (MIC50 52 ± 18 µg/mL) [14]). But, the comparison with the data obtained in our study could not be given due to the difference in extraction method as well as the *S. aureus* strain used. Moreover, Güvenç et al. carried out an antimicrobial study on various extracts from Turkish *Cistus* species by the disc-diffusion method, and found very weak activity of CC and CS aqueous extracts against *S. aureus* ATCC 29,213 and *B. cereus* 1122 strains [44]. In addition, no effect was observed against *P. aeruginosa* and *C. albicans* in this study.

## 4. Conclusions

To our knowledge, this work marks the first study of the phenolic components from aqueous extracts of two Croatian *Cistus* species: *C. criticus* and *C. salviifolius*. In summary, the results of this study of two *Cistus* species from Croatia reveal that both extracts possess prominent antioxidant, antimicrobial, and antiproliferative potential. Preliminary results of cytotoxic activity were obtained and future works have to be done to confirm the safety of these extracts. UPLC-MS/MS data obtained from this study indicate that two *Cistus* extracts are abundant sources of bioactive compounds—polyphenols. Additional studies are also needed to characterize and trace the active compounds with the biological activities of the extracts. The herbal and tea infusions may be an effective auxiliary to prevent or treat cancers, and further study on the precise mechanisms responsible for the antiproliferative activities of these herbal and tea infusions is still required. Altogether, the obtained results highlight the importance of *Cistus* as a promising source of biologically active phytochemical compounds.

## Figures and Tables

**Table 1:** Mass spectrometric data, identification and quantification of phenolic compounds from aqueous extracts of C. creticus L. (CC) and C. salviifolius L. (CS).

Compound	Rt, min	Collision Energy (V)	Ionization Mode	Precursor Ion (m/z)	Fragment Ions (m/z)	Identification	*C. C*. ** (mg/L)	*C. S*. ** (mg/L)
1	1.131	10	−	367	193	Feruloylquinic acid	0.31 ± 0.02	0.07 ± 0.02
2	1.135	10	−	353	119	Caffeoylqunic acid derivative 1	0.16 ± 0.04	0.18 ± 0.28
3	1.169	10	−	324	173	Caffeoylquinic acid derivative 2	0.15 ± 0.06	0.05 ± 0.04
4	1.686	10	−	343	191	Galloylquinic acid	1.16 ± 0.05	5.47 ± 0.49
5	1.693	15	+	442.9	139	Epicatechin gallate *	0.52 ± 0.03	0.23 ± 0.10
6	1.71	10	−	331	169	Monogalloyl glucose [42]	0.66 ± 0.49	2.84 ± 0.22
7	1.758	10	−	169	125	Gallic acid *	2.83 ± 0.11	24.66 ± 0.99
8	2.508	10	−	495	169	Digalloylquinic acid	0.37 ± 0.04	0.33 ± 0.11
9	2.973	15	+	653	303	Quercetinacetylrutinoside	0.41 ± 0.50	0.08 ± 0.01
10	3.993	10		337	163	Coumaroilquinic acid derivative 1	2.48 ± 1.35	0.34 ± 0.47
11	4.014	10	+	579	427	Procyanidin B2 *	11.65 ± 0.31	3.83 ± 0.29
12	4.305	10	+	291	139	Catechin *	5.49 ± 0.26	0.57 ± 0.06
13	4.311	10	+	291	139	Epicatechin *	5.46 ± 0.05	0.52 ± 0.13
14	4.459	10	−	353	119	Chlorogenic acid *	0.06 ± 0.01	0.02 ± 0.01
15	4.974	15	+	597	303	Quercetin-pentosyl-hexoside	0.04 ± 0.01	0.97 ± 0.15
16	5.016	10	−	179	135	Caffeic acid *	0.36 ± 0.55	0.05 ± 0.02
17	5.541	10	−	337	163	Coumaroilquinic acid derivative 2	0.41 ± 0.01	0.39 ± 0.53
18	6.025	15	+	459	139	Epigallocatechin gallate *	0.23 ± 0.06	4.77 ± 0.70
19	6.037	5	+	403	271	Apigenin-pentoside	0.07 ± 0.04	0.03 ± 0.01
20	6.362	5	+	627	315	Myricetin-rutinoside [53]	31.23 ± 0.10	3.09 ± 0.19
21	6.474	15	+	481	319	Myricetin-hexoside [42]	937.44 ± 0.49	564.13 ± 3.78
22	6.571	10	−	163	119	p-coumaric acid *	0.25 ± 0.03	0.23 ± 0.05
23	7.19	15	+	611	303	Ruthin *	3.46 ± 0.06	0.99 ± 0.08
24	7.237	5	+	465	319	Myricetin-rhamnoside [53]	874.51 ± 0.46	3.13 ± 0.71
25	7.254	25	+	319	273	Myricetin *	87.14 ± 0.42	28.27 ± 1.11
26	7.315	10	−	193	134	Ferulic acid *	0.55 ± 0.39	0.07 ± 0.02
27	7.452	5	+	465	303.1	Quercetin-3-glucoside *	26.08 ± 0.20	76.49 ± 1.50
28	7.561	15	+	581	287	Kaempferol-pentosylhexoside	0.03 ± 0.01	0.51 ± 0.11
29	7.65	10	+	647	169	Trigalloylquinic acid	0.24 ± 0.04	0.04 ± 0.02
30	7.971	5	+	495	319	Myricetin-glucuronide	0.76 ± 0.07	0.22 ± 0.08
31	8.044	5	+	435	303	Quercetin-pentoside [54]	6.02 ± 0.02	54.88 ± 1.82
32	8.119	5	+	449	287	Kaempferol-3-glucoside [54]	15.05 ± 0.41	20.56 ± 1.25
33	8.164	15	+	625	317	Isorhamnetin-rutinoside	0.33 ± 0.02	0.03 ± 0.02
34	8.203	35	+	287	153	Luteolin *	4.28 ± 0.23	5.46 ± 1.40
35	8.422	5	+	449	303	Quercetin-rhamnoside [53]	23.02 ± 1.72	0.56 ± 0.29
36	8.651	5	+	419	287	Kaempferol-pentoside	1.14 ± 0.12	5.81 ± 0.79
37	8.723	15	+	637	287	Kaempferol-acetylrutinoside	0.03 ± 0.01	0.05 ± 0.03
38	8.78	10	+	507	303	Quercetin-acetylhexoside	0.13 ± 0.05	0.36 ± 0.56
39	9.541	5	+	491	287	Kaempferol-acetylhexoside	0.05 ± 0.03	0.24 ± 0.15
40	10.610	5	+	433	287	Kaempferol-rhamnoside	1.28 ± 0.07	0.44 ± 0.30
41	10.843	15	+	595	287	Kaempferol-3-rutinoside *	4.56 ± 0.38	12.58 ± 0.80
42	10.846	15	+	595	287	Kaempferol-rhamnosyl-hexoside	7.07 ± 2.89	13.12 ± 1.64
43	11.475	30	+	271	153	Apigenin *	0.35 ± 0.39	0.05 ± 0.03

* Identification confirmed using authentic standards. ** Concentration of individual phenolic compounds are expressed as mg per L of sample, as mean value ± standard deviation.

**Table 2:** Total phenolic content and antioxidant activities of the *Cistus* aqueous extracts.

Antioxidant Assay (units)	*C. creticus*	*C. salvifoliius*	Ascorbic Acid
Folin-Ciocalteu (mg GAE ^a^)	209.27 ± 18.5	161.09 ± 7.2	nd
DPPH ^b^ (mg/mL)	0.52 ± 0.03	0.62 ± 0.04	0.02 ± 0.01
FRAP (equivalents of Fe^2+^ mM ^c^)	0.78 ± 0.02	0.78 ± 0.06	5.57 ± 0.13

^a^ Gallic acid equivalents (mg of GAE/g); ^b^ IC_50_ (mg/mL); ^c^ Sample concentration 1 g/L; Values are represented as mean’s ± SD (*n* = 3).

**Table 3:** Antibiotic resistance profile and genotypic features of the multiple resistant and β-lactamases-producing clinical strains used in this study.

Strain No.	Species	β-lactam Resistance Phenotype	MIC (µg/mL) of Selected Antimicrobial Agents ^a^						β-Lactamase(s) Produced			Non β-Lactam Resistance Phenotype
			AMP	CAZ	IPM	GEN	TET	OX	ESBLs	MBLs	Others	
FSST 02	*Klebsiella pneumoniae*	AMP, SAM, PIP, TZP, TIC, CAZ, CTX, ATM	>1024 (R)	512 (R)	0.5 (S)	8 (R)	128 (R)	NA	SHV-12, CTX-M-15	-	ACC-1, TEM-1	CIP, GEN, TET, SXT
FSST 20	*Acinetobacter baumannii*	AMP, SAM, PIP, TZP, TIC, CTX, CAZ, FEP, ATM, IMP, MEM	>1024 (R)	>256 (R)	16 (R)	16 (R)	64 (R)	NA	-	SIM-1	-	GEN, TOB, AMK
FSST 21	*Pseudomonas aeruginosa*	AMP, SAM, PIP, TZP, TIC, CTX, CAZ, FEP, ATM, IMP, MEM	>1024 (R)	>16 (R)	>8 (R)	>8 (R)	>8 (R)	NA	-	GIM-1	-	CIP, GAT, LVX, AMK, GEN, TOB, NET, TET, SXT
MRSA-1	*Staphylococcus aureus*	OX, P	NA	NA	NA	>16 (R)	<1 (S)	>4 (R)	Modification of PBP *(mecA)*			CC, CIP, E, GEN, MFX

^a^ MIC values were evaluated according to the breakpoints established by CLSI (2012). For *S. aureus* MRSA-1 breakpoint interpretation was done according to EUCAST (2012) guidelines. Abbreviations: R, resistant; S, susceptible; NA, not applicable; AMK, amikacin; AMP, ampicillin; ATM, aztreonam; CAZ, ceftazidime; CC, clindamycin; CIP, ciprofloxacin; CTX, cefotaxime, *E, erythromycin;* FEP, cefepime; GAT, *gatifloxacin;* GEN, gentamicin; IPM, imipenem; LVX, levofloxacin; MEM, meropenem; MFX, *moxifloxacin;* NET, netilmicin; OX, oxacillin; P, penicillin G; PIP, piperacillin; SAM, ampicillin-sulbactam; SXT, trimethoprim-sulfamethoxazole; TET, tetracycline; TIC, ticarcillin; TOB, tobramycin; TZP, piperacillin/tazobactam.

**Table 4:** Antimicrobial activity of the *Cistus* extracts against opportunistic pathogenic bacteria and fungi used in this study.

Species	Strain No.	Inhibition Zone Diameter (mm) ^a^			MIC (µg/mL) ^c^		
		*C. creticus* Extract	*C. salviifolius* Extract	TET ^b^	*C. creticus* Extract	*C. salviifolius* Extract	TET
**Gram-positive bacteria**							
*Staphylococcus aureus*	ATCC 29213	18.4 ± 1.1	15.2 ± 0.9	31.0 ± 0.5	500	1000	0.5 (S)
*Staphylococcus aureus*	MRSA-1	29.0 ± 0.8	25.0 ± 1.0	NT	500	500	<1 (S)
*Bacillus cereus*	FSST-22	13.0 ± 0.5	13.0 ± 0.7	24.6 ± 0.6	1000	1000	1 (S)
*Clostridium* *perfringens*	FSST-24	12.1 ± 0.5	12.4 ± 1.0	NA	1000	500	NA
**Gram-negative bacteria**							
*Klebsiella pneumoniae*	ATCC 13883	17.4 ± 0.5	13.5 ± 1.0	21.3 ± 0.2	1000	1000	1 (S)
*Klebsiella pneumoniae*	FSST-02	10.5 ± 0.0	10.0 ± 0.5	6.0 ± 0.0	2000	2000	128 (R)
*Pseudomonas aeruginosa*	ATCC 27853	12.6 ± 0.9	10.3 ± 0.8	11.5 ± 0.4	1000	1000	32 (R)
*Pseudomonas aeruginosa*	FSST-21	13.2 ± 0.4	11.0 ± 0.5	7.3 ± 0.3	500	1000	>16 (R)
*Acinetobacter baumannii*	ATCC 19606	17.1 ± 0.5	20.5 ± 1.5	17.3 ± 0.6	500	500	2 (S)
*Acinetobacter baumannii*	FSST-20	23.0 ± 0.8	24.2 ± 0.0	6.0 ± 0.0	250	250	64 (R)
				AMB	MIC_90_		AMB
**Yeast**							
*Candida albicans*	FSST-29	19.3 ± 0.8	23.2 ± 1.2	21.6 ± 1.7	500	125	1 (S)

^a^ Diameters of the inhibition zones (in mm) were given for the 6 mm discs impregnated with final concentration of 5 mg/disc of the *Cistus* extracts. All assays were done in triplicate and values expressed as mean ± SD. ^b^ Discs of tetracycline (30 μg) and amphotericin B (10 μg) were used as standards for the disc diffusion assay. ^c^ For fungi, MIC_90_ values represent the lowest concentrations that inhibited 90% of fungal growth when compared to the growth without the addition of extracts. Abbreviations: AMB, amphotericin B; TET, tetracycline; NA, not applicable; NT, not tested; S, susceptible; R, resistant.

## References

[1] Mabberley D.J. (1997). The Plant.

[2] Petropoulos S.A., Karkanis A., Martins N., Ferreira I.C. (2018). Halophytic herbs of the Mediterranean basin: An alternative approach to health. Food Chem. Toxicol..

[3] Papaefthimiou D., Papanikolaou A., Falara V., Givanoudi S., Kostas S., Kanellis A. (2014). Genus Cistus: A model for exploring labdane-type diterpenes’ biosynthesis and a natural source of high value products with biological, aromatic, and pharmacological properties. Front. Chem..

[4] Alkofahi A., Al-Khalil S. (1995). Mutagenic and Toxic Activity of some Jordanian Medicinal Plants. Int. J. Pharmacogn..

[5] Bouamama H., Villard J., Benharref A., Jana M. (2000). Antibacterial and antifungal activities of *Cistus incanus* and C. monspeliensis leaf extracts. Therapie.

[6] Attaguile G., Russo A., Campisi A., Savoca F., Acquaviva R., Ragusa N., Vanella A. (2000). Antioxidant activity and protective effect on DNA cleavage of extracts from *Cistus incanus* L. and *Cistus monspeliensis* L.. Cell Biol. Toxicol..

[7] Rebensburg S., Helfer M., Schneider M., Koppensteiner H., Eberle J., Schindler M., Gürtler L.G., Brack-Werner R. (2016). Potent in vitro antiviral activity of *Cistus incanus* extract against HIV and Filoviruses targets viral envelope proteins. Sci. Rep..

[8] Sayah K., Chemlal L., Marmouzi I., El Jemli M., Cherrah Y., Faouzi M.E.A. (2017). In vivo anti-inflammatory and analgesic activities of *Cistus salviifolius* (L.) and *Cistus monspeliensis* (L.) aqueous extracts. S. Afr. J. Bot..

[9] Wannes W.A., Tounsi M., Marzouk B. (2017). A review of Tunisian medicinal plants with anticancer activity. J. Complement. Integr. Med..

[10] Bereksi M.S., Hassaine H., Bekhechi C., Abdelouahid D.E. (2018). Evaluation of Antibacterial Activity of some Medicinal Plants Extracts Commonly Used in Algerian Traditional Medicine against some Pathogenic Bacteria. Pharmacogn. J..

[11] Jeszka-Skowron M., Zgoła-Grześkowiak A., Frankowski R. (2018). *Cistus incanus* a promising herbal tea rich in bioactive compounds: LC–MS/MS determination of catechins, flavonols, phenolic acids and alkaloids—A comparison with *Camellia sinensis*, Rooibos and Hoan Ngoc herbal tea. J. Food Compos. Anal..

[12] Attaguile G., Perticone G., Mania G., Savoca F., Pennisi G., Salomone S. (2004). *Cistus incanus* and *Cistus monspeliensis* inhibit the contractile response in isolated rat smooth muscle. J. Ethnopharmacol..

[13] Bouamama H., Noel T., Villard J., Benharref A., Jana M. (2006). Antimicrobial activities of the leaf extracts of two *Moroccan cistus* L. species. J. Ethnopharmacol..

[14] Tomás-Menor L., Morales-Soto A., Barrajón-Catalán E., Roldan-Segura C.M., Segura-Carretero A., Micol V. (2013). Correlation between the antibacterial activity and the composition of extracts derived from various Spanish *Cistus* species. Food Chem. Toxicol..

[15] Vitali F., Pennisi G., Attaguile G., Savoca F., Tita B. (2011). Antiproliferative and cytotoxic activity of extracts from *Cistus incanus* L. and *Cistus monspeliensis* L. on human prostate cell lines. Nat. Prod. Res..

[16] Ustun O., Berrin-Ozcelik T.B. (2016). Bioactivities of ethanolic extract and its fractions of *Cistus laurifolius* L. (Cistaceae) and *Salvia wiedemannii* Boiss. (Lamiaceae) species. Pharmacogn. Mag..

[17] Attaguile G. (1995). Gastroprotective effect of aqueous extract of *Cistus incanus* L. in rats. Pharmacol. Res..

[18] Venditti A., Bianco A., Bruno M., Ben Jemia M., Nicoletti M. (2014). Phytochemical study of *Cistus libanotis* L.. Nat. Prod. Res..

[19] Politeo O., Maravić A., Burčul F., Carev I., Kamenjarin J. (2018). Phytochemical Composition and Antimicrobial Activity of Essential Oils of Wild Growing *Cistus* species in Croatia. Nat. Prod. Commun..

[20] Fernández-Calvet A., Euba B., Caballero L., Díez-Martínez R., Menendez M., Ortiz-De-Solorzano C., Leiva J., Micol V., Barrajón-Catalán E., Garmendia J. (2019). Preclinical Evaluation of the Antimicrobial-Immunomodulatory Dual Action of Xenohormetic Molecules against *Haemophilus influenzae* Respiratory Infection. Biomolecules.

[21] Umarusman M.A., Aysan Y., Özgüven M. (2019). Investigation of the antibacterial effects of different plant extracts against pea bacterial leaf blight disease caused by pseudomonas syringae pv. pisi|Farkli Bitki Ekstraktlarinin Bezelye Bakteriyel Yaprak Yanikligina (*Pseudomonas syringae* pv. pisi) A. J. Tekirdag Agric. Fac..

[22] Vasiliki G., Charalampia D., Haralabos K.C. (2019). In Vitro Antioxidant, Antithrombotic, Antiatherogenic and Antidiabetic Activities of *Urtica dioica*, *Sideritis euboea* and *Cistus creticus* Water Extracts and Investigation of Pasta Fortification with the Most Bioactive One. Curr. Pharm. Biotechnol..

[23] Lahcen S.A., El Hattabi L., Benkaddour R., Chahboun N., Ghanmi M., Satrani B., Tabyaoui M., Zarrouk A. (2020). Chemical composition, antioxidant, antimicrobial and antifungal activity of Moroccan *Cistus creticus* leaves. Chem. Data Collect..

[24] Ammendola M., Haponska M., Balik K., Modrakowska P., Matulewicz K., Kazmierski L., Lis A., Kozlowska J., Garcia-Valls R., Giamberini M. (2020). Stability and anti-proliferative properties of biologically active compounds extracted from *Cistus* L. after sterilization treatments. Sci. Rep..

[25] Jerónimo E., Soldado D., Sengo S., Francisco A., Fernandes F., Portugal A.P., Alves S.P., Santos-Silva J., Bessa R.J. (2020). Increasing the α-tocopherol content and lipid oxidative stability of meat through dietary *Cistus ladanifer* L. in lamb fed increasing levels of polyunsaturated fatty acid rich vegetable oils. Meat Sci..

[26] Mahmoudi H., Aouadhi C., Kaddour R., Gruber M., Zargouni H., Zaouali W., Hamida N.B., Nasri M.B., Ouerghi Z., Hosni K. (2016). Comparison of antioxidant and antimicrobial activities of two cultivated cistus species from Tunisia|Comparação das atividades antioxidante e antimicrobiana de duas espécies cultivada de cistus da Tunísia. Biosci. J..

[27] Mikulec A., Kowalski S., Makarewicz M., Skoczylas Ł., Tabaszewska M. (2020). Cistus extract as a valuable component for enriching wheat bread. LWT.

[28] Lisiecka K., Wójtowicz A., Dziki D., Gawlik-Dziki U. (2019). The influence of *Cistus incanus* L. leaves on wheat pasta quality. J. Food Sci. Technol..

[29] Stępień A.E., Gorzelany J., Matłok N., Lech K., Figiel A. (2019). The effect of drying methods on the energy consumption, bioactive potential and colour of dried leaves of Pink Rock Rose (*Cistus creticus*). J. Food Sci. Technol..

[30] Viapiana A., Konopacka A., Waleron K., Wesolowski M. (2017). *Cistus incanus* L. commercial products as a good source of polyphenols in human diet. Ind. Crop. Prod..

[31] Chebli Y., El Otmani S., Chentouf M., Hornick J.-L., Bindelle J., Cabaraux J.F., Chebli Y. (2020). Foraging Behavior of Goats Browsing in Southern Mediterranean Forest Rangeland. Animals.

[32] Guerreiro O., Alves S.P., Soldado D., Cachucho L., Almeida J.M., Francisco A.E., Santos-Silva J., Bessa R.J., Jerónimo E. (2020). Inclusion of the aerial part and condensed tannin extract from *Cistus ladanifer* L. in lamb diets—Effects on growth performance, carcass and meat quality and fatty acid composition of intramuscular and subcutaneous fat. Meat Sci..

[33] Mignacca S.A., Mucciarelli M., Colombino E., Biasibetti E., Muscia S., Amato B., Presti V.D.M.L., Vazzana I., Galbo A., Capucchio M.T. (2019). *Cistus salviifolius* Toxicity in Cattle. Veter. Pathol..

[34] Wittpahl G., Kölling-Speer I., Basche S., Herrmann E., Hannig M., Speer K., Hannig C. (2015). The Polyphenolic Composition of Cistus incanus Herbal Tea and Its Antibacterial and Anti-adherent Activity against *Streptococcus mutans*. Planta Med..

[35] Dimas K., Demetzos C., Marsellos M., Sotiriadou R., Malamas M., Kokkinopoulos D. (1998). Cytotoxic Activity of Labdane Type Diterpenes Against Human Leukemic Cell Lines in vitro. Planta Med..

[36] Raimundo J.R., Frazão D.F., Domingues J.L., Quintela-Sabarís C., Dentinho T.P., Anjos O., Alves M.N., Delgado F. (2018). Neglected Mediterranean plant species are valuable resources: The example of *Cistus ladanifer*. Planta.

[37] El Euch S.K., Bouajila J., Bouzouita N. (2015). Chemical composition, biological and cytotoxic activities of *Cistus salviifolius* flower buds and leaves extracts. Ind. Crop. Prod..

[38] Riehle P., Vollmer M., Rohn S. (2013). Phenolic compounds in *Cistus incanus* herbal infusions—Antioxidant capacity and thermal stability during the brewing process. Food Res. Int..

[39] Rebaya A., Belghith S.I., Cherif J.K., Trabelsi-Ayadi M. (2016). Total phenolic compounds and antioxidant potential of rokrose (*Cistus salviifolius*) leaves and flowers grown in Tunisia. Int. J. Pharmacogn. Phytochem. Res..

[40] El Menyiy N., Al-Waili N., El-Haskoury R., Bakour M., Zizi S., Al-Waili T., Lyoussi B. (2018). Potential effect of *Silybum marianum* L. and *Cistus ladaniferus* L. extracts on urine volume, creatinine clearance and renal function. Asian Pac. J. Trop. Med..

[41] Gürbüz P., Demirezer L.Ö., Güvenalp Z., Kuruüzüm-Uz A., Kazaz C. (2015). Isolation and Structure Elucidation of Uncommon Secondary Metabolites from *Cistus salviifolius* L.. Rec. Nat. Prod..

[42] Gori A., Ferrini F., Marzano M.C., Tattini M., Centritto M., Baratto M.C., Pogni R., Brunetti C. (2016). Characterisation and Antioxidant Activity of Crude Extract and Polyphenolic Rich Fractions from *C. incanus* Leaves. Int. J. Mol. Sci..

[43] Kalpoutzaki E., Aligiannis N., Mitaku S., Harvala C., Skaltsounis L. (2001). New Semisynthetic Antimicrobial Labdane-Type Diterpenoids Derived from the Resin “Ladano” of *Cistus creticus*. Z. Naturforschung C.

[44] Güvenç A., Yıldız S., Özkan A.M., Erdurak C.S., Coskun M., Yılmaz G., Okuyama T., Okada Y., Yildiz S., Yilmaz G. (2005). Antimicrobiological Studies on Turkish *Cistus.* Species. Pharm. Biol..

[45] Garofulić I.E., Zorić Z., Pedisić S., Brnčić M., Dragović-Uzelac V. (2018). UPLC-MS2 Profiling of Blackthorn Flower Polyphenols Isolated by Ultrasound-Assisted Extraction. J. Food Sci..

[46] Abu-Reidah I.M., Ali-Shtayeh M.S., Jamous R.M., Arráez-Román D., Segura-Carretero A. (2015). HPLC-DAD-ESI-MS/MS screening of bioactive components from *Rhus coriaria* L. (Sumac) fruits. Food Chem..

[47] Brand-Williams W., Cuvelier M., Berset C. (1995). Use of a free radical method to evaluate antioxidant activity. LWT.

[48] Benzie I., Strain J. (1996). The Ferric Reducing Ability of Plasma (FRAP) as a Measure of “Antioxidant Power”: The FRAP Assay. Anal. Biochem..

[49] Singleton S., Rossi J.A., Singleton V.L., Singleton V., Rossi J., Singleton V.I. (1965). Colorimetry of total phenolic with phosphomolybdic—Photsphotunstic acid reagents. Am. J. Vitic..

[50] Clinical and Laboratory Standards Institute (2008). Reference Method for Broth Dilution Antifungal Susceptibility Testing of Filamentous Fungi: Approved Standard.

[51] Clinical and Laboratory Standards Institute (2012). Performance Standard for Antimicrobial Susceptibility Testing—Approved Standard.

[52] Maravić A., Skocibusic M., Šamanić I., Fredotović Ž., Cvjetan S., Jutronić M., Puizina J. (2013). Aeromonas spp. simultaneously harbouring blaCTX-M-15, blaSHV-12, blaPER-1 and blaFOX-2, in wild-growing Mediterranean mussel (*Mytilus galloprovincialis*) from Adriatic Sea, Croatia. Int. J. Food Microbiol..

[53] Gürbüz P., Koşar M., Güvenalp Z., Kuruuzum-Uz A., Demirezer L. (2018). Ömür Simultaneous determination of selected flavonoids from different *Cistus* species by HPLC-PDA. J. Res. Pharm..

[54] Barrajón-Catalán E., Fernández-Arroyo S., Roldán C., Guillén E., Saura D., Segura-Carretero A., Micol V. (2011). A systematic study of the polyphenolic composition of aqueous extracts deriving from several *Cistus* genus species: Evolutionary relationship. Phytochem. Anal..

[55] Matłok N., Lachowicz S., Gorzelany J., Balawejder M. (2020). Influence of Drying Method on Some Bioactive Compounds and the Composition of Volatile Components in Dried Pink Rock Rose (*Cistus creticus* L.). Molecules.

[56] Gaweł-Bęben K., Kukula-Koch W., Hoian U., Czop M., Strzępek-Gomółka M., Antosiewicz B. (2020). Characterization of *Cistus × incanus* L. and *Cistus ladanifer* L. Extracts as Potential Multifunctional Antioxidant Ingredients for Skin Protecting Cosmetics. Antioxidants.

[57] Ghalia S., Adawia K., Waed A. (2016). Evaluation of radical scavenging activity, total phenolics and total flavonoids contents of Cistus species in Syria. Int. J. Pharmacogn. Phytochem. Res..

[58] Prior R.L., Wu X., Schaich K. (2005). Standardized Methods for the Determination of Antioxidant Capacity and Phenolics in Foods and Dietary Supplements. J. Agric. Food Chem..

[59] Nicoletti M., Toniolo C., Venditti A., Bruno M., Ben-Jemia M. (2014). Antioxidant activity and chemical composition of three Tunisian Cistus: *Cistus monspeliensis Cistus villosus* and *Cistus libanotis*?. Nat. Prod. Res..

[60] Dudonné S., Vitrac X., Coutière P., Woillez M., Mérillon J.-M. (2009). Comparative Study of Antioxidant Properties and Total Phenolic Content of 30 Plant Extracts of Industrial Interest Using DPPH, ABTS, FRAP, SOD, and ORAC Assays. J. Agric. Food Chem..

[61] Piluzza G., Bullitta S. (2011). Correlations between phenolic content and antioxidant properties in twenty-four plant species of traditional ethnoveterinary use in the Mediterranean area. Pharm. Biol..

[62] Brunetti C., Di Ferdinando M., Fini A., Pollastri S., Tattini M. (2013). Flavonoids as Antioxidants and Developmental Regulators: Relative Significance in Plants and Humans. Int. J. Mol. Sci..

[63] Agati G., Brunetti C., Di Ferdinando M., Ferrini F., Pollastri S., Tattini M. (2013). Functional roles of flavonoids in photoprotection: New evidence, lessons from the past. Plant Physiol. Biochem..

[64] Costa C., Tsatsakis A., Mamoulakis C., Teodoro M., Briguglio G., Caruso E., Sarandi E., Margină D., Dardiotis E., Kouretas D. (2017). Current evidence on the effect of dietary polyphenols intake on chronic diseases. Food Chem. Toxicol..

[65] Friedman M., Mackey B.E., Kim H.-J., Lee I.-S., Lee K.-R., Lee S.-U., Kozukue E., Kozukue N. (2007). Structure−Activity Relationships of Tea Compounds against Human Cancer Cells. J. Agric. Food Chem..

[66] Barrajón-Catalán E., Fernández-Arroyo S., Saura D., Guillén E., Gutierrez A.F., Segura-Carretero A., Micol V. (2010). Cistaceae aqueous extracts containing ellagitannins show antioxidant and antimicrobial capacity, and cytotoxic activity against human cancer cells. Food Chem. Toxicol..

[67] Skorić M., Todorović S.I., Gligorijević N., Janković R., Živković S., Ristić M., Radulović S. (2012). Cytotoxic activity of ethanol extracts of in vitro grown *Cistus creticus* subsp. Creticus L. on human cancer cell lines. Ind. Crop. Prod..

[68] Ismail T., Calcabrini C., Diaz A.R., Fimognari C., Turrini E., Catanzaro E., Akhtar S., Sestili P. (2016). Ellagitannins in Cancer Chemoprevention and Therapy. Toxins.

[69] Lopez-Lazaro M., Calderón-Montaño J.M., Morón E.B., Austin C.A. (2011). Green tea constituents (−)-epigallocatechin-3-gallate (EGCG) and gallic acid induce topoisomerase I- and topoisomerase II-DNA complexes in cells mediated by pyrogallol-induced hydrogen peroxide. Mutagenesis.

[70] Calderón-Montaño J.M., Morón E.B., Pérez-Guerrero C., Lopez-Lazaro M. (2011). A review on the dietary flavonoid kaempferol. Mini Rev. Med. Chem..

[71] Semwal D.K., Semwal R.B., Combrinck S., Viljoen A.M. (2016). Myricetin: A Dietary Molecule with Diverse Biological Activities. Nutrients.

[72] Abad M.J., Bedoya L.M., Bermejo P. (2013). Chapter 14—Essential Oils from the Asteraceae Family Active against Multidrug-Resistant Bacteria A2—Kon, Mahendra Kumar RaiKateryna Volodymyrivna. Fighting Multidrug Resistance with Herbal Extracts, Essential Oils and Their Components.

